# Multiwall Carbon Nanotubes/Spherical Glassy Carbon as Environmentally Friendly Adsorption Materials Utilized in Adsorptive Stripping Voltammetry for the Determination of Trace Amounts of Ga(III)

**DOI:** 10.3390/ma17040966

**Published:** 2024-02-19

**Authors:** Malgorzata Grabarczyk, Marzena Fialek, Edyta Wlazlowska

**Affiliations:** Department of Analytical Chemistry, Institute of Chemical Sciences, Faculty of Chemistry, Maria Curie-Sklodowska University, 20-031 Lublin, Poland; malgorzata.grabarczyk@mail.umcs.pl (M.G.); edyta.wlazlowska@onet.pl (E.W.)

**Keywords:** multiwall carbon nanotubes, spherical glassy carbon, lead film electrode, adsorptive stripping voltammetry, eco-friendly detection of gallium, environmental water samples

## Abstract

This work presents a proposal for an adsorptive stripping voltammetric (AdSV) method for gallium(III) determination at an eco-friendly multiwall carbon nanotube/spherical glassy carbon (MWCNT/SGC) electrode modified with a lead film. The operational factors influencing the sensitivity of the AdSV procedure were thoroughly investigated, and their most favorable values were chosen (0.1 mol L^−1^ acetate buffer solution pH = 5.6; 7 × 10^−5^ mol L^−1^ Pb(II); 2 × 10^−4^ mol L^−1^ cupferron; potential/time of lead film formation: −1.9 V/30 s; potential/time of Ga(III)–cupferron adsorption: −0.75 V/30 s). The newly developed MWCNT/SGCE has proven to be a competitive substrate to the glassy carbon electrode to create a lead film electrode, since it allows the determination of gallium in a wider range of concentrations from 3 × 10^−9^ to 4 × 10^−7^ mol L^−1^ with a lower limit of detection equal to 9.5 × 10^−10^ mol L^−1^. The elaborated procedure has been shown to be highly selective and insensitive to the presence of an even 100-fold excess of most of the ions commonly found in environmental waters. The MWCNT/SGC sensor, which can maintain >95% of its original response after 70 days of use, has been successfully applied for the detection of gallium in water samples with the relative standard deviation (RSD) ranging from 4.5% to 6.2% (*n* = 3) and recoveries in the range from 95.3% to 104.9%.

## 1. Introduction

Stripping voltammetry belongs to the most responsive electroanalytical methods since it involves an electrolytic preconcentration step before the proper electrode process, making the detection limits of this method exceedingly low. A two-step electrochemical measurement permits achieving a limit of detection at the ppb and even sub-ppb magnitude. If the preconcentration step is treated as the outcome of electrolysis, adsorption or electrochemical reaction resulting in the formation of a hardly dissolvable compound on the electrode surface, then we are dealing with anodic stripping voltammetry (ASV), adsorptive stripping voltammetry (AdSV), or cathode stripping voltammetry (CSV), respectively. The adsorption-based method is the most widely used, as this procedure makes it possible to determine metals that cannot accumulate electrolytically [1]. In this method, metal ions can be directly determined by using an appropriate complexing agent that forms an electroactive complex with the element being determined. The complex metal-complexing agent, adsorbed on the electrode in the first stage, undergoes in the stripping stage an oxidation or reduction reaction, resulting in a signal proportional to the concentration of the metal detected. Because a manually selected complexing agent is used, the AdSV method enables the lowest detection limits to be obtained among the above-mentioned voltammetric techniques. Therefore, this approach is most frequently chosen for the determination of different heavy metal ions in various environmental samples, chiefly water samples. This is a significant issue in view of the expansion of the industrial sector, in which heavy metals are used on a large scale. Since a side effect of industrial development is the release of heavy metals into the environment, the content of these metals in various elements of the environment should be constantly checked [2] because heavy metals must be completely removed from waste water [3].

One of the metals most extensively used in industry is gallium. Currently, this element, in the form of a liquid alloy with indium and tin (galinstan), is used as the filling of mercury-free thermometers for measuring body temperature, which are an alternative to mercury glass thermometers withdrawn from production in 2009 due to the harmfulness of mercury to health and the environment [4]. Owing to their semiconductor properties, gallium compounds (notably gallium arsenide (GaAs) and gallium nitride (GaN)) have gained the greatest importance in the electronics industry in the manufacture of optoelectronic devices. They are known to be used in integrated circuits, compact devices, laser diodes, soldering binders, nuclear reactors, and also solar batteries and photodetectors [5,6]. This element is also valued in medicine, as some of its salts are applied in pharmaceuticals and radiopharmaceuticals. Gallium and gallium nitrate(V) are used in the diagnosis and treatment of cancer and diseases of the musculoskeletal system [7]. Gallium is not a highly toxic element, but as a result of its broad usage, its possible undesirable effects on living organisms should be taken into account. The abnormalities that gallium can lead to in humans include the following: enlarged lymph nodes, photophobia, necrosis of lymphatic tissue, kidney damage, or bone marrow diseases. Acute symptoms caused by gallium poisoning in humans involve a metallic taste in the mouth, loss of appetite, drowsiness, nausea, vomiting, skin swelling, exfoliative dermatitis, reduced platelet count, anemia, and bone marrow suppression [8]. In studies conducted on dogs, gallium nitrate(V) was hepatotoxic; after repeated administration after 5 days, the animal’s liver was damaged [9].

According to ISO directives, the recommended method for the determination of Ga(III) in water is inductively coupled plasma mass spectrometry (ICP-MS) [10]. However, due to the cost of the apparatus and thus its unavailability, procedures for the determination of Ga(III) are being developed in many laboratories using other methods. One of such methods is stripping voltammetry, which can be complementary to ICP-MS due to its low instrument cost as well as the speed and simplicity of Ga(III) determination. At the same time, voltammetric procedures provide very low limits of determination often comparable to those obtained with ICP-MS. Literature data indicate that gallium was determined by the adsorption stripping voltammetry method much earlier. However, the first procedures were developed using a hanging mercury drop electrode (HMDE), which is widely known to be toxic in the laboratory environment, as a working electrode [11,12,13,14]. Due to the increased awareness of the harmful effects of mercury, despite the excellent properties of a mercury electrode (such as a wide range of available potentials, perfect polarizability and reproducibility, and the possibility of obtaining very low detection limits), scientists seek to find new environmentally friendly electrode materials that could successfully replace the HMDE electrode. In order to eliminate the use of the HMDE as a source of metallic mercury, the renewable mercury film silver-based electrode (Hg(Ag)FE) was proposed to be used as the working electrode for the determination of Ga(III) [15]. The construction of this sensor substantially reduced the quantity of mercury used for one measurement and restricted its contact with the lab environment [16]. A step forward was made by developing a procedure for gallium determination on a carbon paste electrode (CPE), which completely eliminated mercury [17]. In order to lower the detection limits of gallium, Grabarczyk and Wasag, in turn, decided to use the bismuth film electrode (BiFE) [18], which had been introduced into laboratory work in 2000 by a group of scientists led by Wang [19]. Having successfully developed a more sensitive procedure using the BiFE, the same scientists attempted to determine gallium on a lead film electrode (PbFE) [20] (the PbFE electrode was developed by Korolczuk et al. in 2005 [21]).

Although the procedure [20] has many advantages, unfortunately, it turned out that the sensitivity obtained on the lead film electrode was worse than that on the bismuth film electrode. Hence, the objective of our investigation was to use an environmentally friendly lead film electrode with a simultaneous increase in the sensitivity of Ga(III) quantification. In order to increase the sensitivity of Ga(III) determination, we propose the use of an electrode substrate different than glassy carbon. Therefore, in this work, we focus on replacing glassy carbon as a substrate for forming lead films with a multiwall carbon nanotube/spherical glassy carbon (MWCNT/SGC) electrode, as was completed in the work [20]. This electrode was developed using a mixture of multiwall carbon nanotubes, glassy carbon spherical powder, and epoxy resin. The MWCNT/SGC sensor has already been successfully used as a competitive substrate to the glassy carbon electrode to create bismuth and lead electrodes applied in determination of Cd(II) and Ti(IV), respectively [22,23]. The addition of multiwall carbon nanotubes to spherical glassy carbon makes the novel electrode material demonstrate better performance in comparison with traditional glassy carbon electrodes, improving the sensitivities, dynamic working scope, response time, lifetime, and stability of the nanocomposite sensor due to its high conductivity [22,23]. It has been proven that the incorporation of carbon nanotubes into the electrode material causes a reduction in overpotentials and resistance to surface impurities [24,25,26]. The benefits of using carbon nanotubes as an electrode material result from the unique properties of these structures, such as excellent electrical conductivity, fast electron transfer rate, high adsorption capacity as well as high mechanical strength [27,28,29,30]. A strongly developed specific surface area, which usually ranges from several hundred to several thousand m^2^/g (this means that only 1 g of material would be enough to cover several volleyball courts), is also of great importance [31]. The above-mentioned properties make carbon nanomaterials an excellent material for other applications in many branches of industry and science. Due to their exceptional adsorption capacity and chemical and mechanical properties, multiwall carbon nanotubes have gained global recognition as an environmentally friendly adsorption material. First of all, these carbon structures are used as sorbents adsorbing various types of pollutants on their surface [32]. They are commonly used in filters removing toxic components, capturing gases, and as an element of chemical and environmental sensors used to detect compounds, such as ammonia, oxygen, alcohols, or nitrogen compounds [33,34]. Moreover, the above features determine a wide range of possible applications in photovoltaics; for example, nanomaterials were used in one of the electrodes in solar batteries [35].

Over the years, multiwall carbon nanotubes (MWCNTs) have been commonly used as an electrode material for the determination of both organic compounds, among others nucleic acids [24], hydrazine [25] and tryptophan [26], and metal ions, including Cd(II) [36], Cu(II) [37], Mo(VI) [38] Pb(II) [39], and Ti(IV) [23]. To the best of our knowledge, no study on the determination of Ga(III) by adsorption stripping voltammetric using a MWCNT has been published thus far. Therefore, we focused on developing a new eco-friendly AdSV procedure for the determination of gallium in the form of complexes with cupferron, as in the work [20], but using a working electrode modified with carbon nanotubes. All analytical parameters affecting the signal obtained by the AdSV method were optimized in detail in our procedure and compared with the analytical parameters characterizing the procedure [20] and other procedures based on environmentally friendly electrodes.

## 2. Experimental

### 2.1. Reagents and Chemicals

All studies were carried out using deionized water in a laboratory purification system (Milli-Q system; 18.2 MV; Millipore, Watford, UK). Suprapur or analytical grade chemicals were employed. A standard solution of 1 g L^−1^ of Ga(III) and Pb(II) as well as cupferron were procured from Fluka (Buchs, Switzerland). A working gallium(III) solution was prepared daily to the desired concentrations of 1 × 10^−5^ by dilution with deionized water. Acetate buffers were prepared from acetic acid and sodium hydroxide (Suprapur, Merck, Warsaw, Poland). Multiwall carbon nanotubes (O.D. × I.D. × L 10 nm ± 1 nm × 4.5 nm ± 0.5 nm × 3~6 μm) were purchased from Sigma-Aldrich (St. Louis, MO, USA). Spherical glassy carbon powder, size 0.4–12 µm, was procured from HTW Hochtemperatur-Werkstofe GmbH (Thierhaupten, Germany).

### 2.2. Equipment

All voltammetric measurements were performed on a μAutolab (Eco Chemie, Utrecht, The Netherlands) connected with a personal computer operated by GPES 4.9 software and coupled with a conventional three-electrode system consisting of a multiwall carbon nanotube/spherical glassy carbon (MWCNT/SGC) electrode as a working electrode, an Ag/AgCl (saturated NaCl solution) reference electrode, and a Pt counter electrode. All measurements were carried out using a 10 mL quartz cell. An Orion Star A211 pH benchtop meter (Thermo Scientific, Waltham, MA, USA) was used to measure the pH values of the solutions.

### 2.3. Fabrication of CNT/SGC Electrode

**Figure 1:**
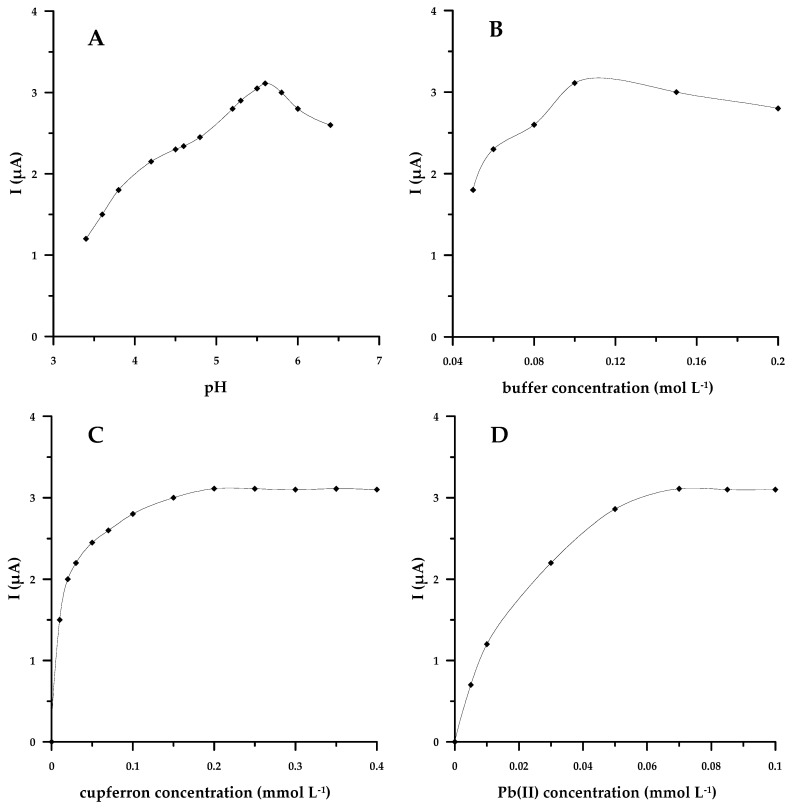
The dependence of the Ga(III) peak current on solution pH (**A**), buffer (**B**), cupferron (**C**), and Pb(II) (**D**) concentration. Concentration of Ga(III) equal to 7 × 10^−8^ mol L^−1^.

Fabrication of the working electrode consisted of mixing MWCNTs with epoxy resin (in a quantitative relation of 1:25) to a homogeneous mass. In the next stage, the created mass was exposed to a temperature of 115 °C and hot centrifuged to expel air bubbles. Subsequently, the resulting mass was mixed with spherical glassy carbon powder (size 0.4–12 µm), maintaining a quantitative ratio of 2:1. The mixture of MWCNTs and SGC obtained in this manner was put under pressure in a casing made of PEEK (polyether ether ketone) in an aperture with a diameter of 2 mm. A copper wire was used to ensure electrical contact. The obtained working electrode was polished on 120 grit sandpaper and then 2000 grit sandpaper. After polishing, the MWCNT/SGC electrode was flushed with large amounts of deionized water and held in an ultrasonic bath (Sonic-3, Polsonic, Warsaw, Poland) for 30 s to remove any remnants of the polishing material. The electrode prepared in the above described way was ready to carry out electrochemical measurements, but before taking measurements, the electrode had to be polished daily with a 0.3 µM suspension of aluminum oxide on a Buehler polishing pad for 30 s, and afterwards, it had to be submerged in an ultrasonic bath for 30 s to remove aluminum oxide remnants. The morphology of the MWCNT/SGC electrode was investigated in the work [22] (see Figure 1 in work [22]). In the above-mentioned paper, the properties of this electrode material were also extensively described.

### 2.4. Procedure for Ga(III) Determination

Standard experiments were performed using differential pulse adsorptive stripping voltammetry (DP-AdSV) in the below described manner. A 10 mL volume of the solution, containing a specific concentration of Ga(III), 0.1 mol L^−1^ acetate buffer pH = 5.6, 2.0 × 10^−4^ mol L^−1^ cupferron, and 7 × 10^−5^ mol L^−1^ Pb(II), were transferred to an electrochemical cell. Under the stirred condition, an accumulation potential of −1.9 V was applied to the electrode for 30 s to create a lead film on the MWCNT/SGC electrode surface. Then, on a freshly formed lead film, the Ga(III)–cupferron complex was adsorbed at a potential value of −0.75 V for 30 s. After stopping the stirrer for 5 s, a negative-trend potential scan, from −0.7 to −1.2 V, was applied at a rate of 100 mV s^−1^, and the peak current of the complex reduction, situated at approximately −0.9 V, was recorded. After each measurement, an electrochemical cleaning process was applied to the electrode at 0.2 V for 15 s under stirring conditions to remove residual metals. The measurements were performed from non-deoxygenated solutions at room temperature. All the results shown in this article were corrected for background. All measurements were performed at room temperature 22 ± 2 °C.

## 3. Results and Discussion

### 3.1. Optimization Studies

The response of the MWCNT/SGC electrode may be affected by all the operational parameters, such as the concentration and pH of the supporting electrolyte, complexing agent concentration as well as the concentration of Pb(II), which is plated directly onto the electrode surface during the analysis. Equally important is the time of the lead film formation and the adsorption of the formed Ga(III)–cupferron complexes on the electrode surface as well as the potential at which these electrochemical processes occur. Bearing this in mind, all these parameters were optimized for the determination of gallium. All optimization studies were carried out at a gallium concentration of 7 × 10^−8^ mol L^−1^.

#### 3.1.1. Dependence on Supporting Electrolyte Composition

As found in previous studies devoted to the determination of gallium in the form of complexes with cupferron, the AdSV peak of gallium was observed only in the presence of CH_3_COOH or CH_3_COOH/CH_3_COONa [14,18,20]. Preliminary tests carried out on an acetate buffer pH = 4.0 and acetic acid confirmed that the MWCNT/SGC electrode shows better sensitivity in an acetate buffer medium. Thus, we decided to use acetate buffer to provide an appropriate analysis environment. When examining the influence of the acetate buffer pH on the gallium signal, it became apparent that the highest current value was obtained at a pH of 5.6. However, both for the lower and higher pH values, the intensity of the peak current tended to decrease (Figure 1A), which is the result of the protonation of cupferron and hydrolysis of gallium, respectively. Hence, the effect of the acetate buffer concentration on the peak current of gallium was studied at pH = 5.6. The concentration of the acetate buffer was altered from 0.05 to 0.2 mol L^−1^. An increase in the gallium signal was observed with increasing buffer concentration up to 0.1 mol L^−1^ (caused by superior buffering at the optimal pH). Nevertheless, at the higher buffer concentrations, the sensitivity decreased (Figure 1B). The reason for this downward trend was most likely the competition of cupferron and acetate, which also occurred in the procedure for the determination of Mo in the form of complexes with cupferron [40]. Consequently, 0.1 mol L^−1^ CH_3_COOH/CH_3_COONa buffer (pH = 5.6) was selected as the supporting electrolyte for the subsequent electrochemical measurements.

#### 3.1.2. Dependence on Complexing Agent Concentration

In order to determine gallium(III) using the proposed AdSV method, it is necessary to introduce cupferron to the voltammetric vessel. To establish what concentration of the complexing agent is the most suitable in our procedure, the height of the analytical signal against the cupferron concentration over the range of 1 × 10^−5^ to 4 × 10^−4^ mol L^−1^ was studied. The sensitivity was found to increase with increasing cupferron concentration, levelling off at complexing agent concentrations above 2 × 10^−4^ mol L^−1^ (Figure 1C). An increase in the concentration of the complexing agent in the voltammetric cell causes more and more gallium ions to be bound, forming electrochemically active complexes, Ga(III)–cupferron, which are then adsorbed on the electrode in the accumulation stage. However, after exceeding the cupferron concentration of 2 × 10^−4^ mol L^−1^, no more cupferron enters into complex bonds with gallium, and an equilibrium is established between cupferron in the form of a complex with gallium and free cupferron, and hence the gallium(III) signal is established at a constant level. In conformity with the acquired data, subsequent measurements were performed at a concentration equal to 2 × 10^−4^ mol L^−1^.

#### 3.1.3. Dependence on Pb(II) Concentration

It should be mentioned that if Pb(NO_3_)_2_ is not added to the analyzed solution, the signal of Ga(III) does not appear in the voltamperogram. This is due to the fact that Ga(III)–cupferron complexes do not adsorb directly on the MWCNT/SGC substrate, and it is necessary to create an intermediate layer, in this case a lead film, to effectively accumulate the Ga(III)–cupferron complex. Therefore, Pb(II) ions are necessary in the analyzed solution to obtain the gallium(III) signal. Moreover, since in our procedure an in situ generated lead film electrode is applied, the thickness of the lead film formed on the electrode substrate depends on the concentration of Pb(NO_3_)_2_ added to the voltammetric cell. At the same time, it was found that the thickness of the lead layer affects the height and shape of the peaks obtained during the determination of metal ions by stripping voltammetry [21]. Therefore, to find the most favorable lead concentration in the test sample, ensuring that the optimal thickness of the lead film is achieved, it was tested how the intensity of the gallium signal differs with growing Pb(II) concentration in the range of 5 × 10^−6^ to 1 × 10^−4^ mol L^−1^. This dependence is as follows: if the lead concentration rises within the range of 5 × 10^−6^–7 × 10^−5^ mol L^−1^, the intensity of the Ga(III) signal tends to increase, whereas for higher lead concentrations, the signal remains unchanged (Figure 1D). Based on the results obtained, it can be concluded that as the Pb(II) concentration increases to 7 × 10^−5^ mol L^−1^, the thickness of the lead layer increases, favoring the adsorption of Ga(III)–cupferron complexes and causing an increase in the Ga(III) signal. However, at a concentration of 7 × 10^−5^ mol L^−1^ Pb(II), the lead film formed has the optimal thickness for the determination of Ga(III), and a further increase in Pb(NO_3_)_2_ concentration does not contribute to an increase in the Ga(III) peak current, which remains constant. Therefore, the Pb(II) concentration of 7 × 10^−5^ mol L^−1^ was adopted as the most optimal for further study.

#### 3.1.4. Potential and Time of Lead Film Formation and Ga(III)–Cupferron Complex Deposition

**Figure 2:**
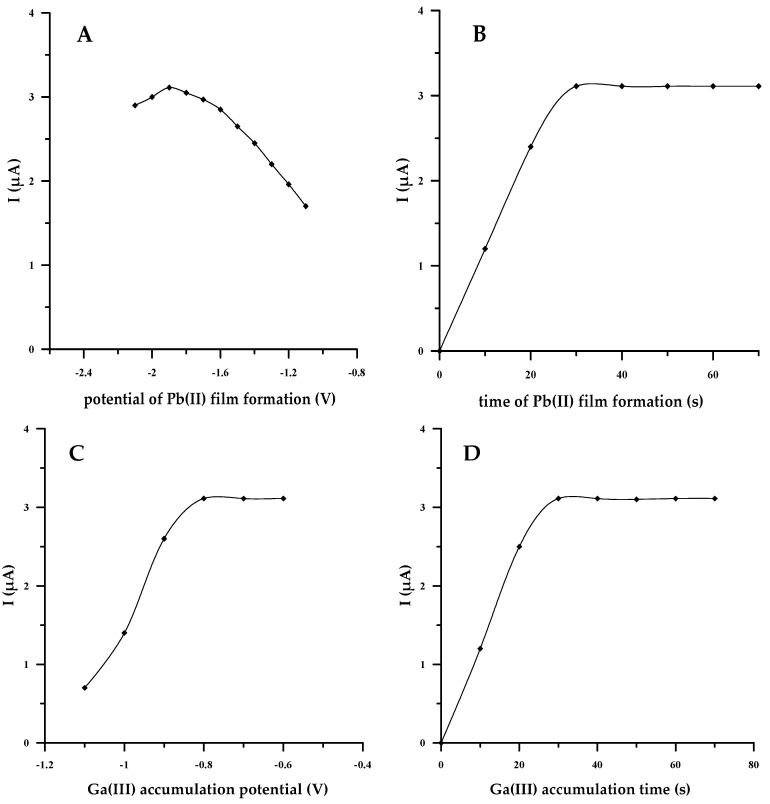
The dependence of the Ga(III) peak current on potential of Pb(II) film formation (**A**), time of Pb(II) film formation (**B**), Ga(III) accumulation potential (**C**) and Ga(III) accumulation time (**D**). Concentration of Ga(III) equal to 7 × 10^−8^ mol L^−1^.

After analyzing the previously developed procedures, we proposed to conduct an in situ measurement to be carried out at two successive potentials: the potential of lead film formation and the potential of gallium adsorption. It was important to determine the deposition potential and time when two-stage studies were undertaken. It is known that these conditions could significantly affect the adsorption of the formed complexes at the working electrode in the AdSV method [14,18,20]. To check it out, the AdSV peak current was investigated for a concentration of gallium equal to 7 × 10^−8^ mol L^−1^ over a potential range from −2.1 to −1.1 V for a fixed time period of 30 s in the first stage, while in the second stage, it was investigated over a potential range from −1.1 to −0.6 V for a fixed time period of 30 s. The data featured in Figure 2 allow us to conclude that the change in the potential of both Pb(II) film formation and Ga(III)–cupferron complex deposition has a significant effect on the voltammetric response of the MWCNT/SGCE toward Ga(III). Therefore, both conditions influence the amount of adsorption of Ga(III) at the electrode. By varying the first potential in the negative direction from −1.1 to −1.9 V, the gallium peak becomes higher and better shaped, while at a more negative potential, the peak becomes broad and much lower. This suggests that the most efficient adsorption of Ga(III)–cupferron onto the lead film occurs when it is formed at a potential of −1.9 V (Figure 2A). A change in the second potential from −1.1 to −0.8 causes an increase in the gallium peak, which stabilizes at a constant level in the range from −0.8 to −0.6 V (Figure 2C). It was found that the most sensitive detection of gallium is possible at the following potential sequence: −1.9 V for 30 s and −0.75 V for 30 s. To optimize the accumulation times, they were changed from 10 to 70 s, with a frequency of 10 s; the obtained results are presented in Figure 2B,D. In both cases, there is a similar upward trend in the gallium peak that grew very quickly with the increase in the accumulation time, which induced the rapid adsorption of Ga(III) on the surface of the modified electrode. The peak current reached the maximum after 30 s and then remained unchanged. This demonstrated that the adsorption on the surface of the modified electrode reached saturation very rapidly. For practical purposes, a 30 s accumulation period was used for the determination of Ga(III).

### 3.2. Reliability of MWCNT/SGCE

Under optimized measurement circumstances, a number of voltammetric measurements were executed to verify the reliability of the MWCNT/SGC electrode. Thus, to look into the repeatability of the sensor, six successive voltammetric quantifications were carried out both for low and considerably high concentrations of Ga(III). The relative standard deviations for the six-fold detection of 5 × 10^−9^ and 1 × 10^−7^ mol L^−1^ were found to be 4.1 and 3.0%, respectively. The reproducibility was determined for the same gallium concentrations as the RSDs according to the experiments executed on seven successive days, and they were 4.7 and 4.5% for the lower and higher concentration, respectively. The long-term stability of the MWCNT/SGC electrode was examined over 70 days at an interval of 7 days. As it turned out, for a period of 70 days, the sensor did not exhibit any significant changes in peak height compared to the signal recorded on the first day. During this time of sensor use, the signal varied at a maximum of ±3%. All these experimental data indicate that the fabricated MWCNT/SGC sensor is reliable and produces consistent and repeatable results.

### 3.3. Standard Work Curve of Ga(III)

**Figure 3:**
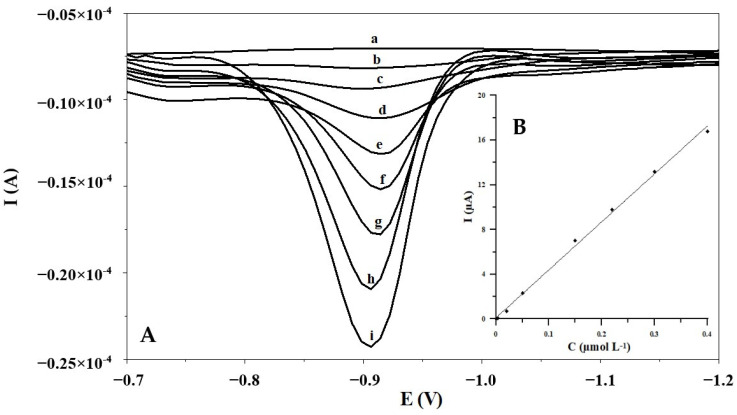
(**A**) The adsorptive stripping voltammograms recorded after preconcentration in different Ga(III) concentrations: (a) 0.0; (b) 0.003 µmol L^−1^; (c) 0.02 µmol L^−1^; (d) 0.05 µmol L^−1^; (e) 0.085 µmol L^−1^; (f) 0.15 µmol L^−1^; (g) 0.22 µmol L^−1^; (h) 0.3 µmol L^−1^; (i) 0.4 µmol L^−1^. (**B**) The plot of the peak current in stripping voltammetry versus the Ga(III) concentration.

The standard work curve was consistent with the following regression equation: y = 42.75x + 0.15, where y is the peak current (μA) and x is the Ga(III) concentration (μmol L^−1^). Each point of the calibration plot is the mean of three values. The correlation coefficient (R^2^) was equal to 0.998. As one can see, the voltammetric response of the fabricated electrode toward Ga(III) increases with increasing Ga(III) concentrations, and there is a good linear relationship between the peak current and Ga(III) concentration in the range from 3 × 10^−9^ to 4 × 10^−7^ mol L^−1^ (Figure 3). On the other hand, the lowest detectable concentrations of Ga(III) absorbed at the in situ plated bismuth film MWCNT/SGC electrode for 60 s are estimated to be 9.5 × 10^−10^ mol L^−1^.

### 3.4. Assessment of Selectivity

In order to examine the selectivity of the MWCNT/SGCE to Ga(III), the sensor was applied for the quantification of 1 × 10^−7^ mol L^−1^ Ga(III) in the presence of 100 times excess of some foreign ions, such as Al(III), As(III), As(V), Bi(III), Ca(II), Cd(II), Cr(III), Cr(VI), Co(II), Cu(II), Fe(III), Mg(II), Mn(II), Ni(II), W(VI), and Zn(II). It was found that a 100-fold excess of Sb(III), Ti(IV) and V(V) decreased the signal of Ga(III) by 17, 42, and 57%, respectively. The most interfering ion proved to be Mo(VI), which caused the complete loss of the Ga(III) signal, even in a 50-fold excess. Other tested ions did not generate any variations in the peak current of more than 5%. For comparison, in the work [20], it was reported that when the GC/PbFE was used, undisturbed measurements were obtained in the presence of a 20-fold excess of Al(III), Bi(III), Ca(II), Cd(II), Cr(III), Cr(VI), Co(II), Cu(II), Fe(III), Mg(II), Mn(II), Ni(II), and Zn(II), but no mention was made of the influence of ions such as Mo(VI), Sb(III), Ti(IV), and V(V). In the procedure [18], in turn, a 100-fold excess of Sb(III), V(V), Mo(VI) and Ti(IV) brought about a reduction in the voltammetric response of the GC/BiFE toward Ga(III) by about 45%, 58%, 66%, and 75%, respectively.

### 3.5. Comparison of Analytical Features of This Approach with Other Eco-Friendly Procedures

The analytical parameters of our procedure in comparison with those of the previously described procedures of gallium determination developed using other environmentally friendly mercury-free electrodes are collated in Table 1. The procedures are ranked in order of increasing detection limit. The presented data confirm our belief that we managed to achieve the intended aim and elaborate a more sensitive procedure using multiwall carbon nanotubes/spherical glassy carbon (MWCNTs/SGC) as the substrate for forming a lead film than the approach [20] using glassy carbon (GC), albeit it is not as sensitive as the methods used by [17,18]. At the same time, the linear response obtained on a substrate modified with carbon nanotubes covers a wider range of gallium concentrations. This metal can be determined in a wider range of its concentrations only using the procedure [18], in which a glassy carbon substrate was covered with a bismuth film. In this work, the linearity range covers 4 orders of magnitude, while the detection limit, equal to 0.1 nmol L^−1^, is the lowest. The procedure [17] also has good sensitivity and allows the determination of gallium concentrations of 0.29 nmol L^−1^, but it is characterized by a relatively narrow range of linearity. On the other hand, the narrowest range of determined gallium concentrations (from 10 to 200 nmol L^−1^) is offered by the method [20] based on a lead film glassy carbon electrode. The method [20] also provides the lowest sensitivity compared to the other ones. Among the procedures based on the film modified working electrode, only in our case the determination of gallium was carried out at two different potential values, corresponding to the deposition of a lead film and the adsorption of the Ga(III)–cupferron complex on the MWCNT/SGC. Moreover, in the procedures based on film electrodes, the accumulation of gallium occurs at a similar potential value due to the use of the same complexing agent, such as cupferron, and the most efficient adsorption of the Ga(III)–cupferron complex is achieved in a relatively short time of 60 s. For comparison, on an unmodified carbon paste electrode, the gallium complex with Alizarin Red S accumulates within 180 s. Moreover, it can be stated that in the developed methods, gallium is determined in an acidic medium, provided by an acetate buffer [17,18] or acetic acid [20].

### 3.6. Analytical Application

**Figure 4:**
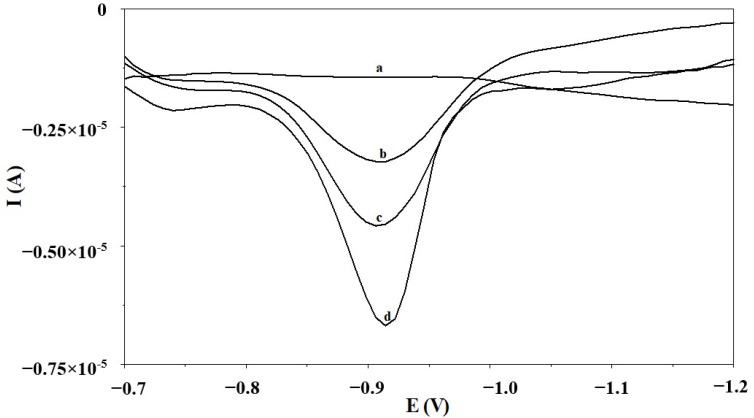
The AdSV voltammogram responses obtained during the gallium analysis in the certified reference material SPS-SW2 Surface Water: (a) SPS-SW2 diluted ten-fold; (b) as (a) + 0.045 µmol L^−1^ Ga(III); (c) as (a) + 0.065 µmol L^−1^ Ga(III); (d) as (a) + 0.110 µmol L^−1^ Ga(III).

To rate the practicability, the obtained MWCNT/SGCE was applied to detect Ga(III) in municipal tap water, river water, and certified reference material, namely SPS-SW2 Surface Water (batch 136). As confirmed by the certificate, SPS-SW2 does not contain gallium but includes 45 trace elements, such as Al, As, B, Ba, Ca, Cd, Ce, Co, Cr, Cs, Cu, Dy, Er, Eu, Fe, Gd, Ho, K, La, Lu, Mg, Mn, Mo, Na, Nd, Ni, P, Pb, Pr, Rb, S, Sc, Se, Si, Sm, Sr, Tb, Th, Tl, Tm, U, V, Y, Yb, and Zn, at a concentration ranging from 2.5 to 10,000 ng mL^−1^. Hence, due to its rich elemental composition, it is a good material for recovery tests. To remove matrix effects, the standard addition method was applied for the analysis of all the above-mentioned real samples. They were analyzed after 10-fold dilution. The results outlined in Table 2 indicate that all of the tested samples are free of Ga(III) and the recoveries calculated for the samples enriched with different amounts of Ga(III) are between 95.3% and 104.9% with the RSD ranging from 4.5% to 6.2%. These results point out that the analytical procedure developed using the MWCNT/SGCE is the right approach to detect Ga(III) in real samples with a complex matrix. Figure 4 illustrates exemplary voltammograms produced during the Ga(III) detection in the certified reference material SPS-SW2 Surface Water.

## 4. Conclusions

This article reports the application of a newly developed environmentally friendly MWCNT/SGC electrode for the determination of Ga(III) by the AdSV method. Based on the experiments performed in this study, the following main conclusions can be drawn about the sensor used:

- It is suitable as a substrate for the formation of a lead film electrode and allows for better sensitivity of gallium determinations compared to the lead film glassy carbon electrode (despite this, the glassy carbon electrode modified with a bismuth film as well as the carbon paste electrode show greater sensitivity);
- Its use allows for a wider range of Ga(III) determination compared to the glassy carbon modified with lead film (simultaneously, it shows a narrower range of Ga(III) determination in comparison with the bismuth film glassy carbon electrode);
- It enables simple and quick detection of Ga(III) with a relatively short single measurement time of 60 s;
- As proven, after 2 months of use, it shows very good stability, as the signal value does not change by more than 3%;
- It was successfully used for the determination of Ga(III) in real samples with a complex matrix and demonstrates a good recovery rate.

## Figures and Tables

**Table 1:** An overview of the procedures based on environmentally friendly mercury-free electrodes used for the determination of Ga(III) by the AdSV method. The procedures are ranked in accordance with increasing LOD.

Working Electrode	Complexing Agent	Supporting Electrolyte	LOD (nmol L^−1^)	Accumulation Potential (V)/ Accumulation Time (s)	Linearity Range (nmol L^−1^)	Ref.
GC/BiFE	Cupferron	Acetate buffer (pH = 4.2)	0.10	−0.65/60	0.30–300	[18]
CPE	Alizarin Red S	Acetate buffer (pH = 4.5)	0.14	−0.05/180	0.29–86	[17]
Pb/MWCNTs/SGC	Cupferron	Acetate buffer (pH = 5.6)	0.95	−1.9/30−0.75/30	3–400	This work
GC/PbFE	Cupferron	Acetic acid	3.8	−0.70/60	10–200	[20]

GC/BiFE—glassy carbon bismuth film electrode; CPE—carbon paste electrode; GC/PbFE—glassy carbon lead film electrode.

**Table 2:** Analytical results of Ga(III) determination in real water samples by means of the AdSV procedure using the MWCNT/SGCE.

Sample	Ga(III) Added (nmol L^−1^)	Ga(III) Found (nmol L^−1^)	Recovery (%)	RSD (*n* = 3) (%)
Tap water	0.0	0.0	-	-
	45.0	46.8	104.0	5.5
	65.0	68.2	104.9	5.1
	110.0	107.8	98.0	4.6
Bystrzyca river water	0.0	0.0	-	-
	45.0	42.9	95.3	6.2
	65.0	63.0	96.9	5.8
	110.0	114.9	104.5	4.9
Certified reference material SPS-SW2 Surface Water	0.0	0.0	-	-
	45.0	43.3	96.2	5.2
	65.0	66.7	102.6	4.7
	110.0	113.5	103.2	4.5

## Data Availability

Data are contained within the article.
